# Implications of labour market disruptions on subjective wellbeing during the COVID-19 pandemic in MENA countries

**DOI:** 10.1016/j.heliyon.2024.e25665

**Published:** 2024-02-12

**Authors:** Mahmoud M. Abdelwahab, Mohamed R. Abonazel, H.E. Semary, Suzan Abdel-Rahman

**Affiliations:** a Department of Mathematics and Statistics, College of Science, Imam Mohammad Ibn Saud Islamic University (IMSIU), Riyadh, 11432, Saudi Arabia; b Department of Basic Sciences, Higher Institute of Administrative Sciences, Osim, Cairo, 12961, Egypt; c Department of Applied Statistics and Econometrics, Faculty of Graduate Studies for Statistical Research, Cairo University, Giza, 12613, Egypt; d Statistics and Insurance Department, Faculty of Commerce, Zagazig University, 44519, Egypt; e Department of Demography and Biostatistics, Faculty of Graduate Studies for Statistical Research, Cairo University, Giza, 12613, Egypt

**Keywords:** COVID-19, Job loss, Income reduction, Subjective wellbeing, MENA countries

## Abstract

COVID-19 has caused a global health crisis and severe economic and social consequences. Unprecedented economic disruption and high unemployment are the pronounced impacts of the pandemic. The current study is primarily concerned with the effects of COVID-19-induced labour market outcomes on workers' subjective wellbeing in four MENA countries using the Combined COVID-19 MENA Monitor Household Survey. The study documented that COVID-19-induced labour market changes negatively affected workers' subjective wellbeing after controlling for work characteristics, risks, social distancing, and socio-demographic variables. Job loss, income reduction, and wage delay were the most significant labour changes that deteriorated workers' subjective wellbeing. Our findings underscore the need for policy responses that reduce workers' vulnerability and sustain their livelihoods. Mental health services and income support policies are important tools to enhance subjective wellbeing of economically affected workers.

## 1 Introduction

The COVID-19 pandemic has affected all aspects of society, including individuals' livelihoods, mental health, and physical health [1]. The COVID-19 outbreak has forced countries to take measures to contain the virus, including the closure of schools, universities, restaurants, and shopping centers; prevention of social gatherings and events; and suspension of airline flights. The pandemic has brought unprecedented changes in the labour market, including rising unemployment, shrinking global growth, and economic crises in most countries.

Throughout the pandemic, many workers lost their jobs and incomes, and others suffered from reduced working hours and wage delays [2]. Even for those who did not lose their jobs, the closure procedures and virus outbreak caused a temporary loss of jobs or furloughs with income losses [3]. The negative implications of the COVID-19 pandemic on employment, income distribution, and food security have significantly affected the individual's well-being [4]. Other restrictions associated with COVID-19 including social distancing and lockdown measures have also severely affected individual's subjective wellbeing [5]. This paper aims to measure the impact of COVID-19-induced labour outcomes on workers' subjective wellbeing in MENA countries based on the WHO-5 questionnaire.

Existing social and economic inequalities have been exacerbated by the crisis. The disproportionate effects of the COVID-19 pandemic on population groups have been widely observed. Low-income and low-skilled workers faced severe economic consequences and were more likely to have reduced working hours and lost their jobs [6]. Low-income and low-educated workers are often informal workers without social insurance coverage and more concentrated in sectors that have been adversely affected by the crisis including food and accommodation, retail, transportation, and home services [7]. Less educated and informal workers were unable to work remotely because their jobs required physical proximity, unlike highly educated and higher-wage workers who were able to work from home [8]. Changes in working hours and wages varied widely across and within countries, depending on employment rates and labour market's characteristics. Developing countries have experienced uneven economic effects due to the pandemic. Governments have not been able to support all workers who were temporarily suspended and were, therefore, more susceptible to infection in their work environment and deterioration of their living conditions. The International Labour Organization (ILO) highlighted that the fiscal stimulus packages needed to compensate for the lost working hours due to the pandemic were 70% lower than required in low-income countries [9], demonstrating how volatile the labour market is in these countries.

Several studies examined the negative effects of unemployment, poverty, and income losses on mental health during economic crises [[10], [11], [12], [13], [14]]. It is well established that unemployment and income losses affect mental health status and other aspects of well-being [[15], [16], [17]]. A causal relationship between socioeconomic status and mental health is also well established [[18], [19], [20], [21]].

Recently, a growing number of studies demonstrated that the COVID-19 pandemic has negatively affected mental health. Depression, anxiety, and poor mental health are common symptoms associated with the COVID-19 pandemic. Davillas and Jones [22] used UK household longitudinal data to investigate psychological distress and found that psychological distress increased by 10% during the pandemic. Daly et al. [23] examined changes in the prevalence of mental health problems before and after the pandemic using the UK household longitudinal study and showed that the prevalence of mental health problems continued to increase significantly during the pandemic and the most psychologically vulnerable groups are young people, females, high educated and high income individuals. Brenner and Bhugra [24] estimated the impact of unemployment and income falls on suicide for both males and females in developed countries and predicated the suicide rates among 38 highly industrialized OECD countries over the period (2000–2017). They found that unemployment increased suicide rate in middle-aged groups while national income losses had a greater impact than unemployment at older ages. Posel et al. [25] investigated the impact of job loss on individuals' mental health in South Africa using data from the National Income Dynamics-Coronavirus Rapid Mobile Survey during 2020 and found that adults who maintained their jobs during the pandemic had lower depression scores than those who lost their jobs, and paid leave increased positively mental health score while unpaid leave had an insignificant effect. Proto and Quintana-Domeque [26] used longitudinal data to measure changes in individuals' mental health during the COVID-19 pandemic, focusing on ethnic groups in the UK, and found a significant difference in mental distress by gender and ethnicity, with white British individuals experiencing the lowest average increase in mental distress. Using data from 11 countries in the Asia-Pacific, Seck et al. [27] assessed how the pandemic affected gender equality in paid and unpaid work and found that women suffered greater loss of employment and worse mental health than men, and their participation rate in unpaid work increased due to caregiving responsibilities. Pieh et al. [28] compared mental health during the pandemic lockdown and 6 months later using six mental health indicators including depression, anxiety, sleep quality, perceived stress, quality of life, and wellbeing, and found that the detrimental consequences of the pandemic on mental health indicators persisted for a long time after the pandemic outbreak. Nkire et al. [29] also estimated the impact of COVID-19-related lockdown measures on mental health and found that self-isolation and quarantine significantly increased depression, stress, and anxiety.

Some studies measured the consequences of COVID-19 on mental health focusing on a specific population group. For example, Xie et al. [30] investigated students' mental health in home confinement during the pandemic in Hubei province, China, and found that they had increased and unexpected symptoms of depression and anxiety. Kivi [31] used measures of anxiety, risk perception, and social distancing to investigate the effects of the pandemic on four aspects of older adults' well-being (life satisfaction, financial satisfaction, self-rated health, and loneliness), and found that adults' well-being remained stable or even increased compared to previous years. Giuntella et al. [32] used longitudinal data to compare the lifestyle and mental health of young adults before and after the COVID-19 pandemic and to identify depression's drivers. They found higher rates of depression and large disruptions to sleep, time use, physical activity, social interactions, and mental health during the pandemic, showing that physical activity disruption is the key factor in experiencing depression Haque et al. [33] focused on the mental health status of informal waste workers in Bangladesh and found that workers who experienced COVID-19 symptoms, lost their incomes, and had to reduce their daily meals were more likely to suffer from psychological distress.

Despite the huge global literature, the impact of the COVID-19 pandemic on mental health and well-being in the MENA region has been documented in a limited number of studies. Giovanis and Ozdamar explored the types of coping strategies taken by individuals and households to deal with income losses during the pandemic and their impact on subjective well-being in four Arab countries, the study found that the costs of the adopted coping strategies differ significantly across coping strategies and countries [34]. Barsoum and Majbouri focused on the impact of COVID-19 on women's work, care work, and subjective well-being (SWB) in four countries in the MENA region and found that women's unemployment rate rose significantly during the pandemic and the main determinants of their well-being are household income and time spent on housework [35].

Although the pandemic has had serious repercussions on workers' economic conditions and their mental health is expected to worsen due to lockdown measures and disrupted labour market in the MENA region, to date, no study has investigated the impact of COVID-19- induced labour market outcomes on workers' subjective well-being in MENA countries. It is critical to understand how the pandemic and its associated implications have affected workers' well-being. In this context, the current study aims to investigate how COVID-19-induced labour market outcomes have shaped workers' subjective well-being in the MENA region. In particular, the study assesses the degree to which experiencing negative labour outcomes, including job loss, reduced working hours, change of main activity, and income decline have affected the workers' subjective well-being in four MENA countries, namely Egypt, Morocco, Jordan, and Tunisia. Our study contributes to previous research in two aspects. First, it provides evidence on subjective well-being status and its link with labour market outcomes during the COVID-19 pandemic. Second, our evidence for MENA countries adds to available evidence which has drawn mainly on foreign countries.

## 2 Data and methods

### 2.1 Data

The study used the latest available data collected from MENA countries. The study is primarily based on the Combined COVID-19 MENA Monitor Household Survey (CCMMHH) collected by the Economic Research Forum (ERF). CCMMHH are phone surveys covering a nationally random sample of mobile users aged 18–64. CCMMHH provides integrated harmonized data for Arab countries. It investigated the effects of COVID-19 on the labour market, income and earnings, mental health, food security, coping strategies, social safety nets, social distancing, and online education. It provided detailed information about employment status of individuals and induced negative changes in their incomes, jobs, mental health status. It included different modules for workers, women, farmers, and enterprises. It covered the period from November 2020 to August 2021 through five bimonthly rounds. The total number of respondents is 29,818 distributed as follows (Tunisia = 8143, Morocco = 8120, Jordan = 7625, and Egypt = 5930). Available data provides four waves for Tunisia and Morocco and three waves for Jordan and Egypt. CCMMHH is a wide-ranging and representative at the national levels. The sample was selected using three attempts of random digit dialing. Sampling procedures and non-response are considered in weights, for more details about the sampling procedure, response rates, and weights, see Ref. [36]. Informal consent was obtained from all participants at the beginning of the survey. All participants were informed about the purpose of the survey and data confidentiality.

### 2.2 Methodology

Subjective wellbeing (SWB) was measured using the WHO-5 short questionnaire. WHO-5 consists of five questions that mainly focus on positive dimension of mental health. Respondents were asked how often in the last two weeks they felt cheerful and in good spirits; calm and relaxed; active and vigorous; refreshed and rested upon waking; And about whether their daily lives are full of things that interest them. The five questions were measured on a Likert scale ranging from 0 to 5 so that higher scores indicate better subjective wellbeing. Response options were “at no time”, “some of the time”, “less than half the time”, “more than half the time”, “most of the time”, “and all the time”. Responses were then aggregated to provide an overall assessment of subjective wellbeing. The WHO-5 is a valid questionnaire and widely used in previous studies. It showed acceptable consistency based on our dataset, where the Cronbach's alpha is 0.82 for Egypt, 0.85 for Morocco, 0.75 for Tunisia and 0.79 for Jordan. The overall The Cronbach's alpha across the four countries is 0.79. Independent samples *t*-test and analysis of variance (ANOVA) F-test are used to compare subjective wellbeing scores across groups.

Workers were asked about negative changes in the labour market during the pandemic. They were asked whether, in the last 60 days, they faced any of the following outcomes due to COVID-19 or related restrictions: reduction in working hours, decline in income, delays in wage payment, temporary layoff, permanent layoff, inability to work from home, and change in the main activity. These outcomes are non-mutually exclusive choices. Hierarchical multiple regression models were estimated in which subjective wellbeing score is regressed on labour market outcomes after hierarchical control for other variables [37]. The full model is

$${SWB}_{ijt}=b_{0}+b_{1}{LMO}_{ijt}+b_{2}{WC}_{ijt}+b_{3}X_{ijt}+b_{4}C_{j}+b_{5}W_{t}+\varepsilon_{ijt}$$

where ${SWB}_{ijt}$ refers to the subjective wellbeing score for worker i in country j and wave t. i=1,2,…,N; j=1,2,3,4; t=1,2,3,4,5. ${LMO}_{ijt}$ denotes the labour market outcome experienced by the workers i during the pandemic. Each outcome takes a value of 1 if encountered by the worker and 0 otherwise. ${WC}_{ijt}$ refers to work characteristics before the pandemic including occupation, sector type, work stability, social insurance, and work inside establishment. $X_{ijt}$ include worker characteristics (age, gender, educational level, marital status, place of residence, household size, presence of children, and household income quartile). Social distancing and risks are also included in X. Risks include levels of worry about catching COVID-19 and economic situation, while social distancing variables include staying at least 1 m away from others, wearing a mask, and washing hands more often than before COVID-19. $C_{j}$ is the country-fixed effect, $w_{t}$ is the wave- fixed effect. $\varepsilon_{ijt}$ is the error term. We limited the data to respondents working before the pandemic to estimate the effects of negative labour outcomes on their subjective wellbeing. The total number of workers is 10,013 workers (Tunisia = 3100, Morocco = 2519, Jordan = 2642, and Egypt = 1752). The definition of each variable in detail is presented in Table A1 in the appendix.

The analysis was performed through a set of sequential steps. We checked the assumptions of linear regression using residuals analysis. We checked the linearity of the data (response-independent relationships) through examining the residuals versus the fitted values. Homogeneity of the residual variance was assessed using scale location plot which showed equally spread points for some countries. Heteroskedasticity robust standard errors are provided. Outlying values were detected using residuals versus leverage plot. We deleted any outliers whose standardized residuals exceed 3 standard deviations. We used Cook's distance metric to detect any influential points that could affect the model results. Multicollinearity was assessed by computing the variance inflation factor (VIF) to assure that all independent variables have VIF less than 5 [55]. These steps are performed in the final model in each country. Performance metrics (Adjusted R-squared, AIC, BIC) were estimated to select the best model and the lower the AIC and BIC metrics, the better the model [56,57].

## 3 Results

### 3.1 Subjective wellbeing status of workers

More than three-quarters of the workers were males (78.3%), the majority were adults (69.6%), currently married (60.8%) and urban residents (73.5%). One-third of the workers were educated up to the basic level (33.4%) and about 35% had a higher education level. Among the workers, 16.9 % belonged to the first income quartile. Clerks/service workers represented 30.6% of total sample and 26.3% were blue-collar, agricultural, production, and transport workers. Workers in economic activities “manufacturing”, “construction or utilities”, and “retail or wholesale” are the most representative in data. Before the COVID-19 pandemic, 74.3% of these workers were working in the private sector, 48.7% were uninsured, 62% were engaged in irregular work (causal, seasonal, or intermittent), and 36.9% were employed outside the establishments. Table 1 presents the demographic and socio-economic profile of the wage workers.

**Table 1:** Characteristics of workers and their wellbeing subjective scores.

Worker characteristics	Mean ± SD	Test statistic & P-value	N (%)
**Gender**			
Male	10.17 ± 6.26	t=3.94	9883 (78.3)
Female	9.74 ± 5.77	<0.001***	2737 (21.7)
**Place of residence**			
Urban	9.99 ± 6.20	t=0.393	9275 (73.5)
Rural	10.35 ± 6.07	0.652	3322 (26.3)
**Age**			
Younger adults <30	10.23 ± 6.34	F=*8.53* <0.001***	3578 (28.4)
adults (30–59)	9.99 ± 6.07		8780 (69.6)
older adults >60	11.54 ± 7.12		262 (2.1)
**Marital status**			
Never married	10.28 ± 6.33	F=*9.06* <0.001***	3533 (28.0)
Currently married	10.03 ± 6.06		7677 (60.8)
Widowed/divorced	9.20 ± 6.64		367 (2.9)
**Educational level**			
Less than basic education	9.52 ± 6.34	F=22.71 <0.001***	2131 (16.9)
Basic education	10.04 ± 6.57		2081 (16.5)
Secondary education	10.53 ± 6.05		4009 (31.8)
Higher education	10.53 ± 5.68		4399 (34.9)
**Household income quartile**			
1st quartile	9.57 ± 6.69	F=*21.25* <0.001***	1959 (16.9)
2nd quartile	9.96 ± 6.29		3332 (28.8)
3rd quartile	10.13 ± 5.87		3401 (29.4)
4th quartile	10.73 ± 5.67		2887 (24.9)
**Number of children**			
0	10.18 ± 6.21	F=*1.92* 0.147	6717 (58.0)
1–2	10.01 ± 6.41		4391 (37.9)
3+	9.70 ± 6.34		469 (4.1)
**Occupation before the pandemic**			
Blue collar, production, and transport	9.31 ± 5.67	F=*29.13* <0.001***	3040 (26.3)
Clerks &Service workers	10.11 ± 6.55		3544 (30.6)
Technicians &Associate professionals	10.51 ± 6.24		2839 (24.5)
Managers& Professional	10.83 ± 5.97		2155 (18.6)
**Main economic activity before the pandemic**			
Agriculture, fishing & mining	10.29 ± 6.19		773 (6.1)
Manufacturing	10.25 ± 6.33		1616 (12.8)
Construction & utilities	9.90 ± 6.27		1578 (12.5)
Retail & Wholesale	9.85 ± 6.09		1576 (12.5)
Transportation & storage	8.47 ± 5.73		1070 (8.5)
Accommodation &food services	9.82 ± 6.41		931 (7.4)
Information & communication	10.36 ± 6.44		449 (3.6)
Financial activities &real estate	10.88 ± 5.90		660 (5.2)
Education	10.13 ± 5.43		1421 (11.3)
Health	9.87 ± 5.67	F=4.504	694 (5.5)
Other services	10.90 ± 6.52	<0.001***	1852 (14.7)
**Type of sector**			
Public sector	10.68 ± 5.96	t=7.42	3238 (25.7)
Private sector	9.91 ± 6.26	<0.001***	9382 (74.3)
**Social insurance status**			
Insured	10.36 ± 6.07	t=5.95	6473 (51.3)
Uninsured	9.79 ± 6.25	<0.001***	6147 (48.7)
**Employment stability**			
Irregular (causal, seasonal, or intermittent)	9.33 ± 6.09	t=9.92	3747 (29.7)
Regular (permanent or temporary)	10.46 ± 6.17	<0.001***	7830 (62.0)
**Work inside establishments**			
Yes	10.35 ± 6.04	t=6.93	7306 (63.1)
No	9.66 ± 6.34	<0.001***	4271 (36.9)
**Able to work remotely during the pandemic**			
Yes	10.44 ± 5.89	t=3.72	2253 (20.9)
No	10.15 ± 6.22	<0.001***	8526 (79.1)

**Note**: ***P < 0.001, **p < 0.01, *p < 0.05.

Table 1 also provides mean and standard deviation of subjective wellbeing score according to workers characteristics. Adult workers were at higher risk of poor SWB, while SWB scores of younger or older adults were less affected by the pandemic. There was significant gender gap in SWB scores in favour of males, the average SWB scores for males exceeded those for females by 0.54 points. SWB scores did not differ significantly between urban and rural residents. Widowed/divorced workers had worse SWB than married or never married workers. There were significant differences in SWB scores across the educational levels and income quartiles. The relationship between the number of children and SWB had an insignificant negative trend. Work characteristics were highly associated with SWB. SWB scores varied significantly by occupation with blue-collar, agricultural, production, transport, and storage workers having the lowest SWB score. SWB scores were substantially lower among workers in “transportation and storage” and “accommodation and food services” activities than workers in other economic activities. SWB was worse among workers who were uninsured and can't work remotely. Workers in irregular work and in the private sector experienced significantly lower SWB scores.

### 3.2 Labour market outcomes and corresponding subjective wellbeing scores during the pandemic

COVID-19 has disrupted the MENA labour market and created many negative outcomes. More than half of workers experienced substantial changes in their employment status. As indicated in Table 2, 19.7% of workers lost their jobs temporarily, 9% lost their jobs permanently and 22.3% experienced a reduction in their working hours. 16.8% of workers saw their income decline and 21% got paid late. On the contrary, 80.8% were able to maintain their same hourly wages before COVID-19 and a small proportion not exceeding 3% earned higher incomes during the pandemic.

**Table 2:** Distribution of workers according to labour market changes and wellbeing scores.

Labour market changes	Mean ± SD	Test statistic & P-value	N (%)
**Temporary Layoff/suspension of Work**			
Mentioned	9.09 ± 5.69	t=−8.66	2187 (19.7)
Not mentioned	10.50 ± 6.23	<0.001***	8923 (80.3)
**Permanent layoff/suspension of work**			
Mentioned	8.87 ± 6.01	t=−7.39	1001 (9.0)
Not mentioned	10.36 ± 6.16	0.001**	10,109 (91)
**Changes in working hours**			
Decline in working hours	9.29 ± 5.36	F=*7.67* <0.001***	2713 (22.3)
No change in working hours	10.41 ± 6.25		8795 (72.4)
Increase in working hours	9.53 ± 5.71		465 (5.3)
**Changes in hourly wage**			
Decline in income	8.67 ± 5.21	F=24.47 <0.001***	2047 (16.8)
Stable income	10.45 ± 5.98		9817 (80.8)
Increase in income	10.98 ± 6.31		289 (2.4)
**Delay in wage payment**			
Mentioned	8.90 ± 5.24	t=−10.52	2337 (21.0)
Not mentioned	10.55 ± 6.33	<0.001***	8773 (79.0)
**Change in main job/activity**			
Different	9.14 ± 6.34	t=−6.85	1116 (13.8)
The same	10.56 ± 6.25	<0.001***	6984 (86.2)
**No changes in employment status**			
Mentioned	11.00 ± 4.25	t=11.96	5374 (48.4)
Not mentioned	9.49 ± 5.87	<0.001***	5736 (51.6)

***P < 0.001, **p < 0.01, *p < 0.05.

Experiencing different labour market outcomes vary significantly by pre-pandemic work characteristics. Informal workers have suffered disproportionately from job and income losses. Uninsured and private-sector workers reported negative labour outcomes more than insured and public-sector workers. Regular workers are more subjected to permanent layoffs, wage delays, and income reductions than irregular workers. The same findings were found for workers inside establishments. Blue-collar, skilled agricultural, production, and transport workers experienced large-scale layoffs and substantial reductions in their income. Also, workers in “construction or utilities” activities suffered from negative changes in labour market more than those in other activities, for more details see, Table A2.

COVID-19 changes in the labour market differed significantly across MENA countries. About 30 % of Tunisian workers temporarily lost their jobs compared to only 9% of Jordanian workers. 32.5% of Tunisian workers had wage delays compared to only 8.9% of Moroccan workers. The highest percentage of permanently laid-off workers was in Egypt (10.4%), while the lowest percentage was in Jordan (8.2%). Additionally, Egyptian workers were more exposed to reduced working hours and wage cuts (40% and 30.9%, respectively). In contrast, most Moroccan workers did not change their employment status and maintained their regular wages and working hours during the pandemic (63.3%, 92.4% and 80.9%, respectively), see Table A3.

Negative labour market outcomes could explain the current variation in workers' SWB scores. Subjective wellbeing of workers who experienced income reduction or job loss is expected to be worse than workers who maintain their jobs, incomes, and usual working hours. As expected, the results revealed an obvious gap in SWB scores between workers who did and did not experience negative labour outcomes. Table 2 showed that workers who maintain their employment status had a higher average SWB score reaching 11 points. While temporarily laid-off workers had lower SWB scores than those who maintain their jobs by 1.4 points. Mean SWB score was also lower among permanently laid-off workers than those who kept their jobs during the pandemic. Permanently laid-off workers had lower SWB scores on average than those temporarily laid off. Workers whose working hours did not change during the pandemic had higher SWB scores than those experiencing increased or reduced working hours. In addition, workers who had received higher incomes reported higher SWB scores than those with reduced or stable incomes. Delaying wages and changing main were also associated with lower SWB scores.

**Fig. 1:**
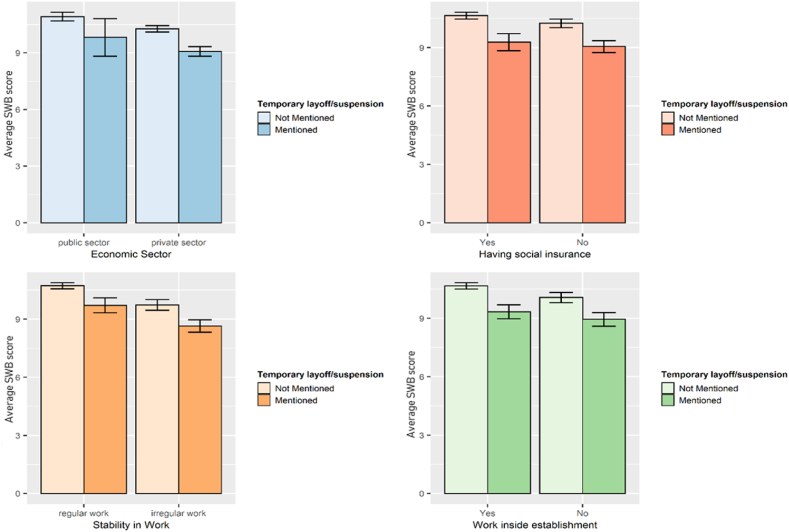
Average SWB of temporarily laid-off workers according to their work characteristics.

**Fig. A1:**
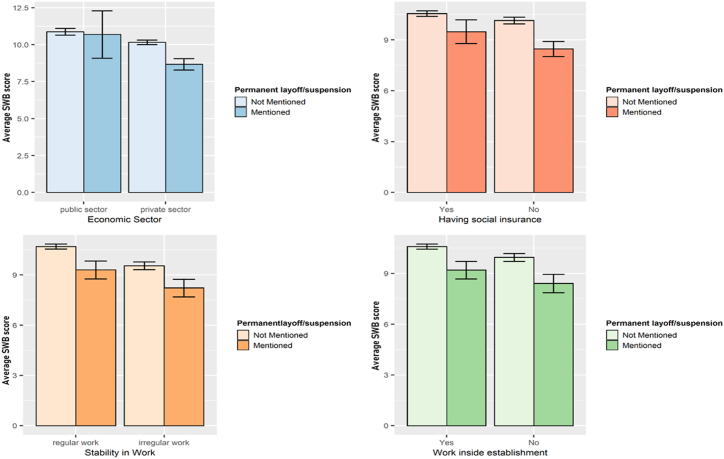
Average SWB score of permanently laid-off workers by pre-pandemic work characteristics.

**Fig. A2:**
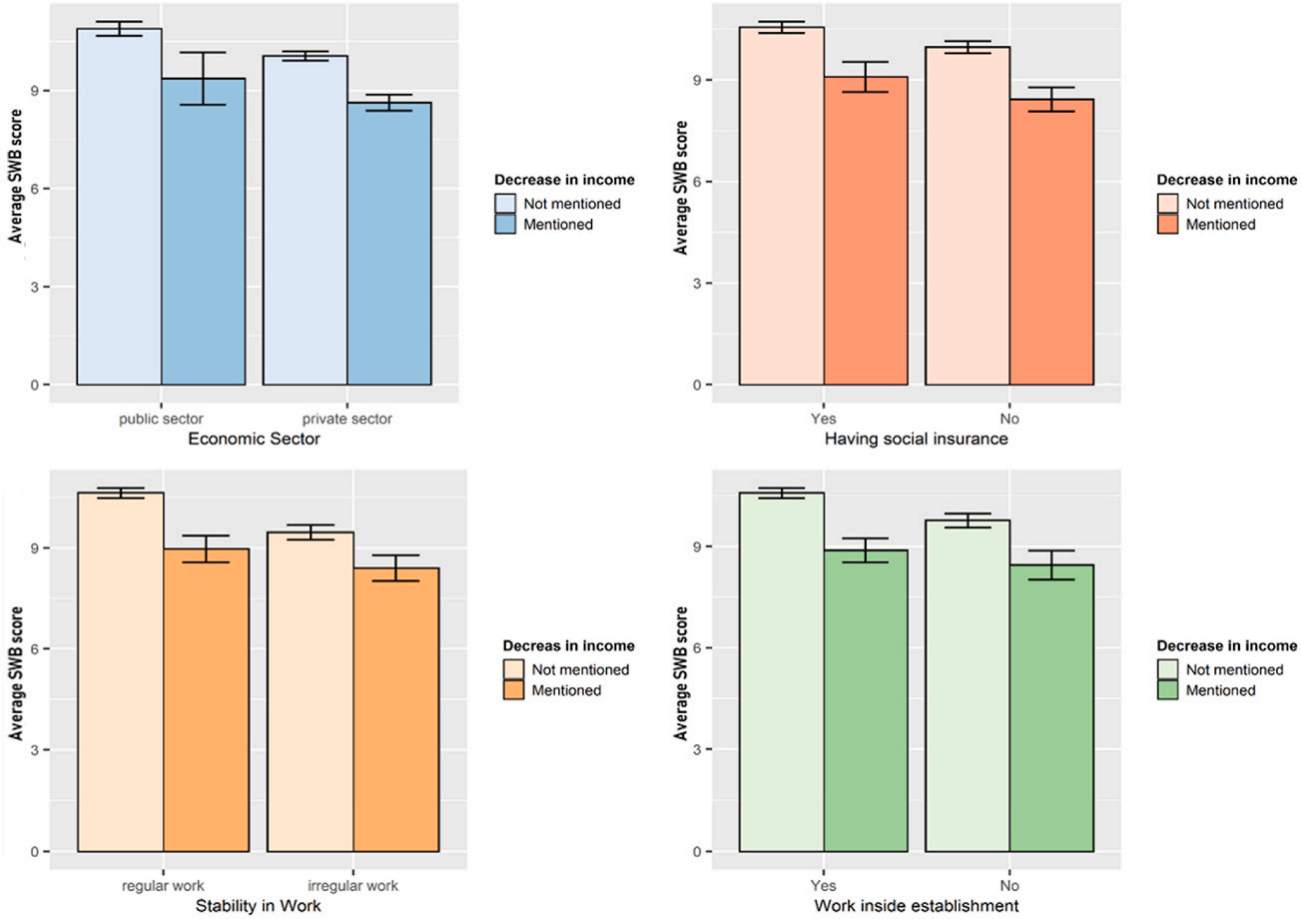
Average SWB score of workers experiencing income fall by pre-pandemic work characteristics.

Pre-COVID-19 work characteristics limited the impact of negative labour changes on workers’ subjective wellbeing. As depicted in Fig. 1, among workers who suffered temporary layoff, those who belonged to the public sector, had social insurance, worked in regular jobs and inside establishments had higher mean SWB scores than those in the private sector, without health insurance, worked in irregular jobs and outside establishments. Similar results are found among workers who experienced permanent layoff and income reduction, see Figs. A1 and A2 in the appendix.

**Fig. A3:**
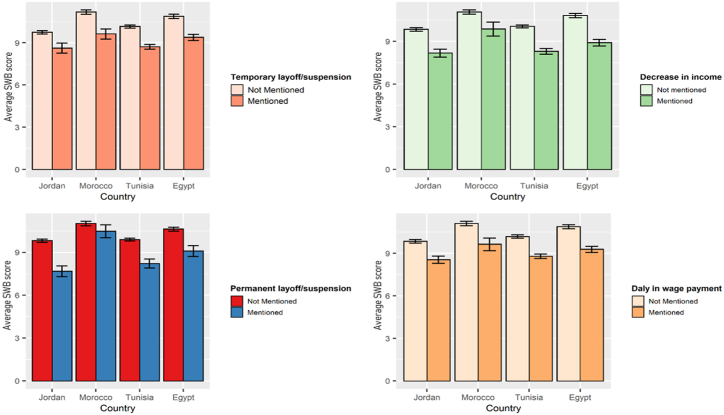
Average SWB score of workers in MENA countries according to negative labour market outcomes.

Subjective wellbeing scores also differed significantly across countries. Jordanian workers had the lowest SWB score while Moroccan workers have the highest score. The impact of negative labour outcomes on SWB scores holds across all countries. Workers who experienced negative labour outcomes such as temporary or permanent layoff, reduced working hours, incomes declines, and wage delays had lower SWB scores than those who did not mention these negative outcomes, Fig. A3.

**Fig. A4:**
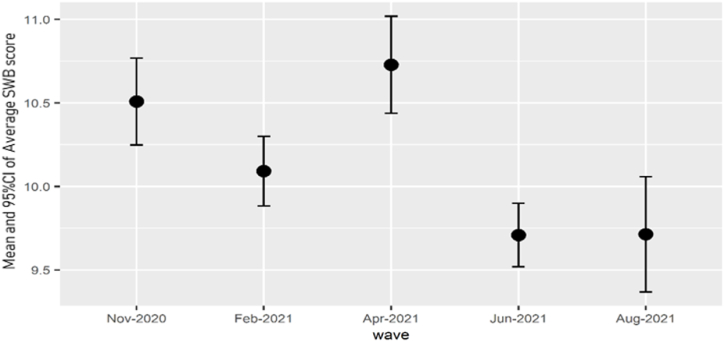
Average SWB and 95% confidence interval across COVID-19-waves.

SWB scores did not improve over time, average SWB decreased from 10.51 points in the first wave to 10.09 points in the second wave when workers began experiencing the negative effects of the pandemic on jobs, incomes, and living standards. Although the average SWB score increased in the third wave to 10.73 points, it decreased directly to 9.71 points in the fifth wave, Fig. A4.

**Fig. A5:**
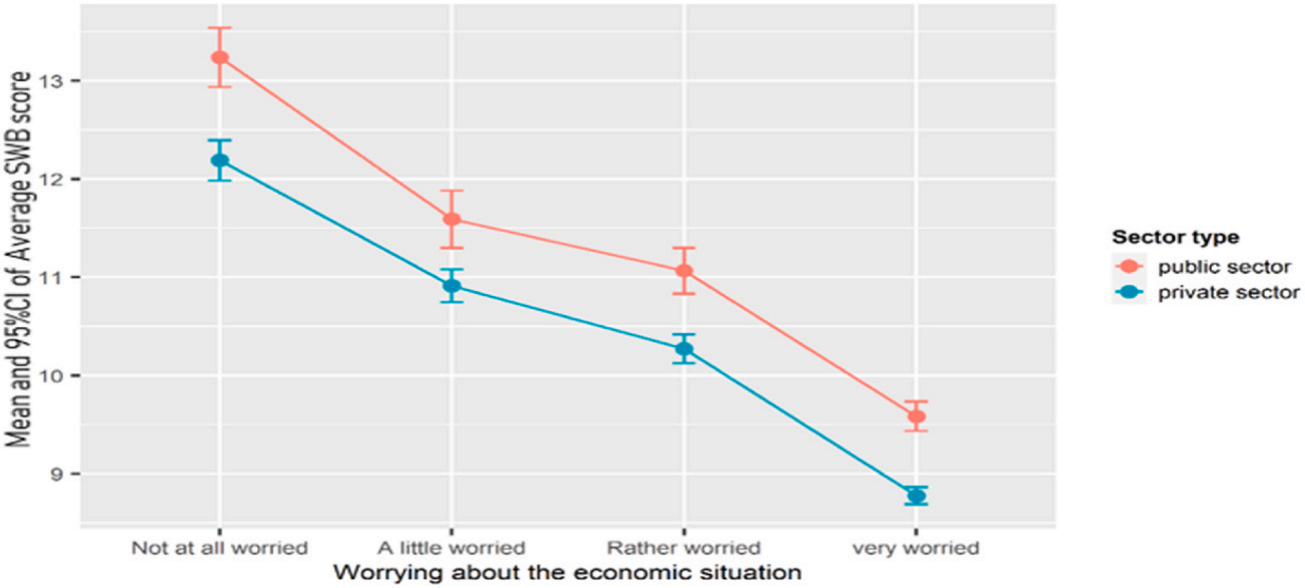
Mean and confidence interval of SWB score by level of worrying about economic situation and sector type.

**Fig. A6:**
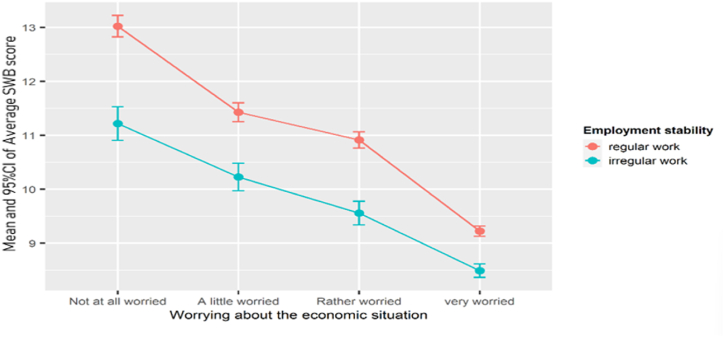
Mean and confidence interval of SWB score by level of worrying about economic situation and employment stability.

**Fig. A7:**
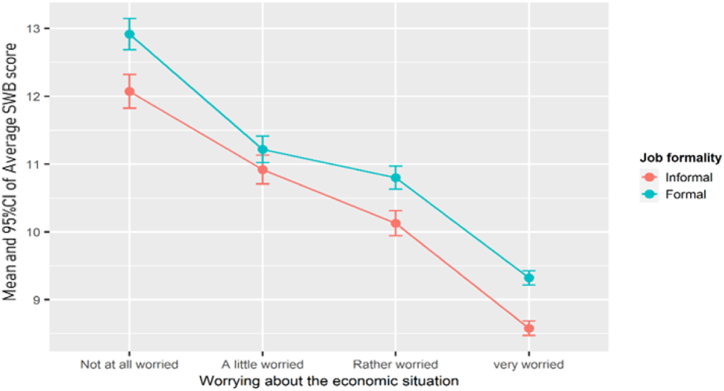
Mean and confidence interval of SWB score by level of worrying about economic situation and job formality.

**Fig. A8:**
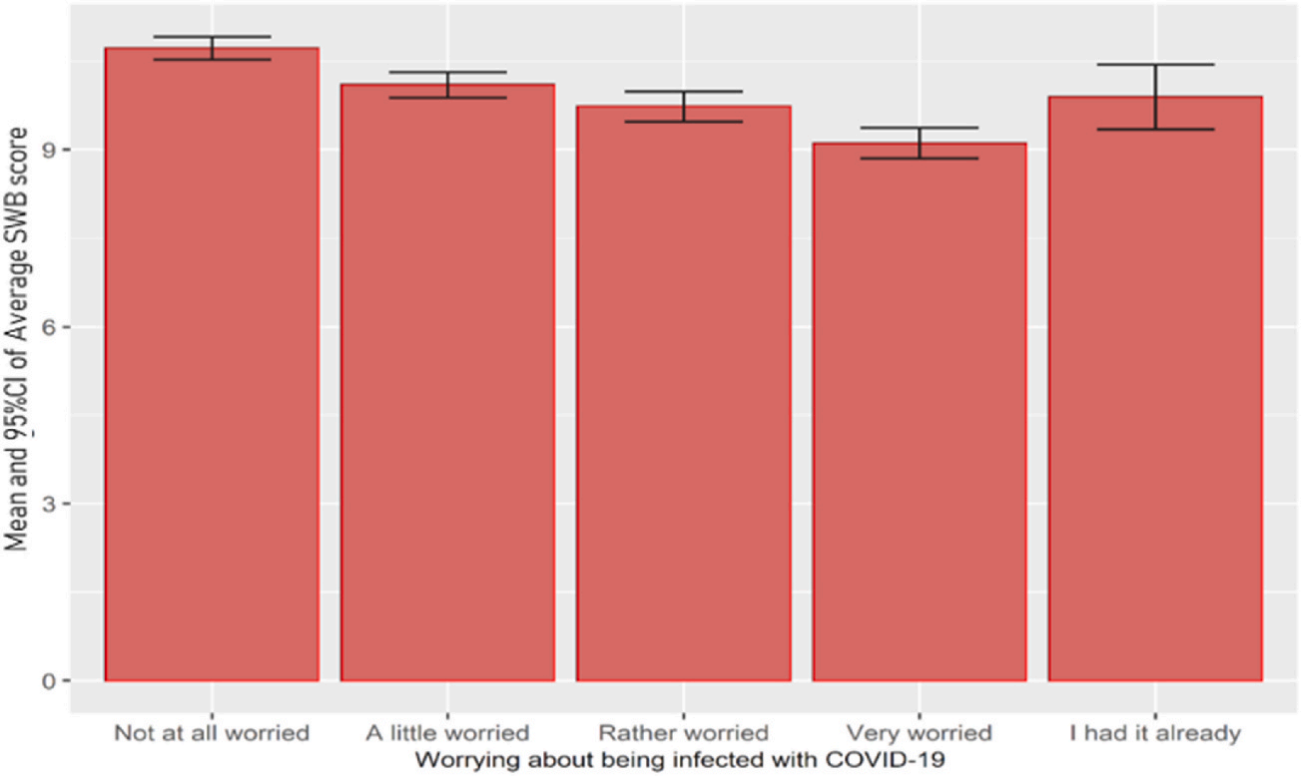
Mean and confidence interval of SWB score by level of worrying about being infected with COVID-19.

Uncertainty conditions and low-income expectations negatively affected subjective wellbeing during the pandemic. SWB scores were the lowest among workers very worried about their economic situation [8.97 points] and the highest among workers not worried at all [12.51 points]. The impact of worrying about the economic situation on SWB varied according to work characteristics. SWB scores of workers in the private sector, irregular, and informal jobs were the worst at all levels of worry, as displayed in Figs. A5–A7. In addition, worrying about catching COVID-19 negatively affected worker's subjective wellbeing. Workers very worried about infection have the lowest average SWB score [9.11 points], while workers not worried at all have the highest score [10.72 points], Fig. A8.

### 3.3 Hierarchical multiple regression models

This section provides results of the hierarchical multiple regression models. Table 3 presents four statistical models: Model (1) measured the impact of labour market changes on subjective wellbeing. Model (2) estimated the impact of labour market changes after controlling for pre-COVID-19 work characteristics, including work regularity, job formality, sector type, occupation, and economic activity. Model (3) considered the potential effects of risks and social distancing variables, while Model (4) controlled for the demographic characteristics. All models included fixed effects for country and wave variables. The results demonstrated the negative impact of labour market changes on workers' subjective wellbeing. Workers who experienced temporary or permanent layoffs during the pandemic had lower SWB scores by 1.09 points and 0.67 points than their counterparts who kept their jobs, respectively. Workers' subjective wellbeing was not significantly affected by reducing working hours and not allowing work from home. While income cuts and wage delays had negative impacts on SWB scores, with a decrease of 1.01 and 1.09 points. SWB scores for workers who changed their main job/activity also decreased significantly by 1.11 points. Even after controlling work characteristics, risks, social distancing, and demographic variables, negative labour outcomes still had significant effects on workers' subjective wellbeing except for temporary job loss.

**Table 3:** The hierarchical multiple regression of subjective wellbeing status during the COVID-19 pandemic.

Variables	Model (1)	Model (2)	Model (3)	Model (4)
**COVID-19 work outcomes**				
Temporary Layoff/suspension	−0.669** (0.196)	−0.416* (0.205)	−0.316 (0.204)	−0.346 (0.211)
Permanent Layoff/suspension	−1.086 ***(0.251)	−0.972 ***(0.253)	−0.971***(0.246)	−0.933***(0.269)
Decline in working hours	−0.252 (0.201)	−0.159 (0.200)	−0.066 (0.198)	−0.106 (0.206)
Decline in income	−1.011***(0.219)	−0.926***(0.192)	−0.749**(0.249)	−0.726**(0.229)
Delay in wage payment	−1.086*** (0.191)	−0.998***(0.192)	−0.834***(0.192)	−0.770 ***(0.199)
unable to work from home	−0.215 (0.185)	−0.088 (0.198)	−0.052 (0.193)	−0.070 (0.204)
Change in main job/activity	−1.107 ***(0.238)	−0.881***(0.242)	−0.762**(0.237)	−0.774** (0.244)
**Work characteristics before COVID-19**				
Working in Public sector		0.091 (0.223)	−0.072 (0.219)	−0.044 (0.230)
Having social insurance		−0.017 (0.181)	0.026 (0.177)	0.139 (0.186)
Having regular work		0.848***(0.186)	0.689***(0.183)	0.620**(0.190)
Working inside establishment		0.747***(0.196)	0.875***(0.187)	0.786***(0.199)
**Occupation**				
Blue collar, production & transport workers		−0.828** (0.268)	−0.800**(0.262)	−0.564*(0.293)
Clerks/service workers		−1.031***(0.234)	−0.925***(0.229)	−0.823***(0.244)
Technicians/associate professionals		−0.353 (0.244)	−0.312 (0.239)	−0.242 (0.249)
Manager/professional (**reference)**				
**Main economic activity**				
Agriculture, fishing & mining **(reference)**				
Manufacturing		−1.144**(0.409)	−1.026*(0.400)	−1.061*(0.427)
Construction & utilities		−0.330 (0.405)	−0.249 (0.396)	−0.254 (0.417)
Retail &Wholesale		−1.028 *(0.413)	−0.884*(0.405)	−0.922*(0.429)
Transportation & storage		−1.196 **(0.434)	−1.053*(0.426)	−1.143**(0.450)
Accommodation &food services		−1.405**(0.442)	−1.144**(0.433)	−1.226**(0.461)
Information & communication		−1.492** (0.551)	−1.200*(0.539)	−1.397*(0.569)
Financial activities & real estate		−0.817 (0.541)	−0.668 (0.525)	−0.803 (0.535)
Education		−1.523***(0.445)	−1.173**(0.435)	−0.994*(0.439)
Health		−1.616 ***(0.476)	−1.278**(0.466)	−1.117*(0.502)
Other services		−1.100**(0.427)	−0.979*(0.416)	−0.978*(0.475)
**Risks**				
**Worrying about economic situation**				
Not at all worried **(reference)**				
A little worried			−1.490***(0.285)	−1.451***(0.293)
Rather worried			−1.659***(0.282)	−1.618***(0.291)
Very worried			−3.068***(0.249)	−2.984***(0.257)
**Worrying about catching COVID-19**				
Not at all worried **(reference)**				
A little worried			−0.797*** (0.206)	−0.772***(0.214)
Rather worried			−0.804 ***(0.225)	−0.731** (0.247)
Very worried			−1.071***(0.227)	−0.908***(0.236)
I had it already			−1.024**(0.373)	−1.007**(0.379)
**Social distancing controls**	No	No	Yes	Yes
**Demographic controls**	No	No	No	Yes
**Waves fixed effects**	Yes	Yes	Yes	Yes
**Country fixed effects**	Yes	Yes	Yes	Yes
**Constant**	11.24 ***(0.353)	13.24*** (0.565)	13.48***(0.698 (	13.96*** (0.828)
**Adjusted R-squared**	0.033	0.044	0.083	0.087
**AIC**	42,854	42,793	42,525	40,570
**BIC**	42,963	43,017	42,818	40,974

Note: Regression models are estimated using OLS. Parentheses display heteroskedasticity robust standard errors.

***P < 0.001, **p < 0.01, *p < 0.05.

As indicated in Model (2), pre-pandemic work characteristics are significantly correlated with workers' subjective wellbeing. The results indicated that regular jobs and work inside establishments were protective factors against deteriorating subjective wellbeing. The unexpected results are that work in the public sector and health insurance coverage did not significantly impacted workers' SWB scores. Occupation and economic activity played significant roles in shaping workers' wellbeing. The SWB scores for categories “Blue collar, production and transport workers” and “Clerks/service workers” decreased significantly by 0.83 and 1.03 points. Workers in most economic activities had lower SWB scores than those working in “Agriculture, fishing or mining” activities. Health and education workers recorded the highest drops in SWB scores with 1.62 and 1.52 points. As shown in models (3) and (4), the impact of work characteristics persisted with unsubstantial changes in coefficient's estimates after controlling other variables: risks, social distancing, and demographic characteristics.

Risks created by COVID-19 were also core drivers of subjective wellbeing status during the pandemic. The more the worker worries about the economic situation, the worse his subjective wellbeing. Workers very anxious about their economic situation experienced a significant deterioration in their SWB scores by 3.06 points compared to those not worried at all (reference group). The same pattern was observed for workers worried about contracting COVID-19 infection, very worried workers about catching the virus had lower SWB scores than those not worried at all by 1.07 points. Risk factors generally remained significant after controlling income level and other socio-demographic variables. Adding risk variables to Model 3 improved performance metrics in contrast to adding demographic variables to Model 4. The best model that had the smallest AIC and BIC and the highest adjusted R-squared was Model 4.

The study measured the impact of labour market outcomes on workers' subjective wellbeing in each country separately, Table 4, Table 5, Table 6, Table 7. Results showed that all labour outcomes changes had significant negative effects on the subjective wellbeing of Egyptian workers, except for the temporary job loss and reduced working hours. A change in the main activity during the pandemic was the most contributing factor with 1.3 points drop in SWB scores of Egyptian workers. While permanent job losses and wage cuts reduced Tunisian workers' SWB scores by 1.41 and 1.28 points, respectively. Wage delays were responsible for reducing Moroccan workers’ SWB by 1.35 points, respectively. Lastly, the decrease in income reduced the SWB scores of Jordanian workers by 0.96 points.

**Table 4:** The hierarchical multiple regression of subjective wellbeing status during COVID-19 in Egypt.

Variables	Model (1)	Model (2)	Model (3)	Model (4)
**COVID-19 work outcomes**				
Temporary Layoff/suspension	−0.721*(0.341)	−0.727*(0.355)	−0.501 (0.347)	−0.395 (0.351)
Permanent Layoff/suspension	−0.903 (0.474)	−0.995*(0.477)	−0.962*(0.467)	−0.983*(0.467)
Decline in working hours	−0.662 (0.339)	−0.439 (0.349)	−0.318 (0.339)	−0.294 (0.340)
Decline in income	−0.893*(0.408)	−1.011*(0.412)	−0.751 (0.403)	−0.863*(0.405)
Delay in wage payment	−1.037**(355)	−1.001**(0.358)	−0.803*(0.350)	−0.741*(0.350)
Unable to work from home	−0.524 (0.341)	−0.683 (0.443)	−0.689*(0.343)	−0.856*(0.349)
Change in main job/activity	−1.488***(0.441)	−1.363**(0.443)	−1.316**(0.434)	−1.258**(0.443)
**Work characteristics before COVID-19**	No	Yes	Yes	Yes
**Risks**	No	No	Yes	Yes
**Social distancing controls**	No	No	Yes	Yes
**Demographic controls**	No	No	No	Yes
**Waves fixed effects**	Yes	Yes	Yes	Yes
**Constant**	12.085***(0.356)	13.85***(0.814)	15.28***(0.956)	16.33***(1.168)
**Adjusted R-squared**	0.045	0.064	0.112	0.120
**AIC**	9098	9084	9017	9020
**BIC**	9151	9227	9213	9300

**Note**: Regression models are estimated using OLS. Parentheses display heteroskedasticity robust standard errors. ***P < 0.001, **p < 0.01, *p < 0.05.

**Table 5:** The hierarchical multiple regression of subjective wellbeing status during COVID-19 in Tunisia.

Variables	Model (1)	Model (2)	Model (3)	Model (4)
**COVID-19 work outcomes**				
Temporary Layoff/suspension	−0.651 (0.341)	−0.392 (0.370)	−0.198 (0.361)	−0.369 (0.408)
Permanent Layoff/suspension	−1.559***(0.464)	−1.393**(0.477)	−1.289**(0.464)	−1.412**(0.527)
Decline in working hours	0.406 (0.372)	0.437 (0.375)	0.663 (0.367)	0.635 (0.423)
Decline in income	−1.095*(0.428)	−1.036*(0.434)	−1.243**(0.422)	−1.275*(0.494)
Delay in wage payment	−0.908**(0.335)	−0.762*(0.339)	−0.485 (0.333)	−0.222 (0.383)
unable to work from home	0.136 (0383)	0.383 (0.397)	0.528 (0.387)	0.666 (0.448)
Change in main job/activity	−0.033 (0.466)	0.039 (0.473)	−0.018 (0.462)	0.306 (0.519)
**Work characteristics before COVID-19**	No	Yes	Yes	Yes
**Risks**	No	No	Yes	Yes
**Social distancing controls**	No	No	Yes	Yes
**Demographic controls**	No	No	No	Yes
**Waves fixed effects**	Yes	Yes	Yes	Yes
**Constant**	11.029***(0.392)	11.032***(0.787)	13.32***(0.960)	12.57***(1.34)
**Adjusted R-squared**	0.032	0.034	0.091	0.089
**AIC**	9535	9548	9466	7543
**BIC**	9599	9703	9674	7823

Note: Regression models are estimated using OLS. Parentheses display heteroskedasticity robust standard errors. ***P < 0.001, **p < 0.01, *p < 0.05.

**Table 6:** The hierarchical multiple regression of subjective wellbeing status during COVID-19 in Morocco.

Variables	Model (1)	Model (2)	Model (3)	Model (4)
**COVID-19 work outcomes**				
Temporary Layoff/suspension	−1.218*(0.534)	−1.132* (0.536)	−1.085*(0.529)	−0.961 (0.528)
Permanent Layoff/suspension	−0.328 (0.658)	−0.709 (0.656)	−0.688 (0.647)	−0.715 (0.647)
Decline in working hours	−1.243*(0.615)	−0.959 (0.612)	−0.728 (0.606)	−0.693 (0.606)
Decline in income	−0.379 (0.780)	−0.587 (0.776)	−0.170 (0.766)	−0.183 (0.763)
Delay in wage payment	−1.368*(0.723)	−1.577*(0.719)	−1.408*(0.709)	−1.345*(0.708)
unable to work from home	0.332 (0.403)	0.556 (0.424)	0.450 (0.418)	0.335 (0.433)
Change in main job/activity	−1.363*(0.554)	−0.788 (0.561)	−0.794 (0.557)	−0.870 (0.559)
**Work characteristics before COVID-19**	No	Yes	Yes	Yes
**Risks**	No	No	Yes	Yes
**Social distancing controls**	No	No	Yes	Yes
**Demographic controls**	No	No	No	Yes
**Waves fixed effects**	Yes	Yes	Yes	Yes
**Constant**	11.28***(0.457)	12.47***(1.208)	15.06***(1.501)	15.58***(1.68)
**Adjusted R-squared**	0.014	0.038	0.071	0.082
**AIC**	12,237	12,208	12,158	12,151
**BIC**	12,452	12,372	12,367	12,303

**Note**: Regression models are estimated using OLS. Parentheses display heteroskedasticity robust standard errors. ***P < 0.001, **p < 0.01, *p < 0.05.

**Table 7:** The hierarchical multiple regression of subjective wellbeing status during COVID-19 in Jordan.

Variables	Model (1)	Model (2)	Model (3)	Model (4)
**COVID-19 work outcomes**				
Temporary Layoff/suspension	−0.235 (0.484)	0.277 (0.499)	0.175 (0.487)	0.300 (0.489)
Permanent Layoff/suspension	−1.678**(0.532)	−1.236*(0.537)	−1.096*(0.524)	−0.906 (0.526)
Decline in working hours	0.111 (0.425)	0.137 (0.427)	0.027 (0.416)	0.051 (0.417)
Decline in income	−1.532**(0.477)	−1.396**(0.481)	−1.033*(0.471)	−0.962*(0.472)
Delay in wage payment	−1.147**(0.429)	−0.887*(0.439)	−0.780 (0.428)	−0.756 (0.429)
unable to work from home	−0.781*(0.336)	−0.195 (0.401)	0.104 (0.394)	0.311 (0.407)
Change in main job/activity	−1.208**(443)	−0.9117*(0.451)	−0.539 (0.441)	−0.691 (0.443)
**Work characteristics before COVID-19**	No	Yes	Yes	Yes
**Risks**	No	No	Yes	Yes
**Social distancing controls**	No	No	Yes	Yes
**Demographic controls**	No	No	No	Yes
**Waves fixed effects**	Yes	Yes	Yes	Yes
**Constant**	11.299***(0.345)	9.254***(1.143)	9.145***(1.36)	10.06***(1.61)
**Adjusted R-squared**	0.034	0.047	0.097	0.105
**AIC**	11,756	11,748	11,658	11,659
**BIC**	11,817	11,902	11,867	11,957

Note: Regression models are estimated using OLS. Parentheses display heteroskedasticity robust standard errors. ***P < 0.001, **p < 0.01, *p < 0.05.

## 4 Discussion

The pandemic has affected lifestyle and mental health in unprecedented ways. Our study highlighted the predictive power of labour market changes in explaining the workers' subjective wellbeing during the pandemic. Previous studies have documented that employment status played an important role in shaping workers' subjective wellbeing; employed were happier and more satisfied with their lives than the unemployed [10,11,15,16]. On the contrary, growing unemployment and large-scale layoffs during the pandemic could mitigate the negative impact of unemployment because it is a general situation for most workers. Some studies suggested that the impact of unemployment on subjective wellbeing s was less severe during global economic crises because it was beyond an individual's control which reduces the stigma associated with unemployment [38]. However, the current study found that most labour market outcomes retain their significance in shaping workers' subjective wellbeing during the pandemic even after controlling potential confounders.

Deterioration in subjective wellbeing is the largest among workers experiencing permanent layoff/suspension. This finding is consistent with other studies that reported that individuals who maintained their jobs are less likely to experience depression symptoms than those who lost their jobs [13,25,[39], [40], [41]]. In line with previous studies, wage delays and income losses were correlated with poor mental health [33,42]. Cotofan et al. [1] found that furloughed workers without wages losses had low levels of well-being compared to those who maintained their jobs during the pandemic. Barsoum and Majbouri [35] and Donnelly and Farina [43] also found that households members who experienced income shocks due to job loss were more likely to develop symptoms of anxiety and depression. While Zhou et al. [44] argued that the relationship between work outcomes and individuals' mental health went beyond unemployment and working hours reduction and temporary job loss have worsened workers' mental health more than unemployment. The current study also found that workers who had to change their main activity suffer from poor mental health during the pandemic.

Working from home is one of the salient changes triggered by the pandemic in the working environment. Home workers are deprived of building relationships with their managers and co-workers which reduces their job satisfaction. Bloom et al. (2014) found that the well-being of workers who lost their jobs was negatively affected by the loss of social connection, daily routine, and job-related identity [45]. Giuntella et al. [32] also found that disrupting physical activity was a significant determinant of depression during the pandemic, indicating that individuals who have succeeded in maintaining their lifestyle had better mental health. On the other hand, telecommuting was a key factor in maintaining employment during the pandemic. Many benefits have been achieved from accomplishing work from home including keeping jobs and reducing infection in crowded workplaces [6]. However, our study indicated that inability to work from home had only significant effect in Egypt and negatively affected workers’ subjective wellbeing.

Our findings indicated that pre-COVID-19 work characteristics contributed to the observed variability in SWB scores. Regular workers had better SWB scores than irregular worker. Workers inside establishment also had better SWB scores. Unexpected results were that the government sector and social insurance coverage did not protect workers against the negative impacts of COVID-19. These findings are consistent with previous studies showing that the negative impacts of COVID-19 were more substantial for private-sector workers than for public sector workers and informal workers have been the hardest hit by the pandemic in MENA countries [46,47]. Cajner et al. [48] also discussed that most public sector workers have permanent employment contracts protecting them from layoffs and income losses during crises and that public sector has furloughed infected workers, reduced working hours, and enabled others to work remotely without any reduction in income.

Despite the unequal repercussions of the pandemic on workers across different economic sectors, the subjective wellbeing of most workers has been negatively affected. Workers in economic activities related to accommodation and food services, transportation and storage, and retail and wholesale suffered from worse subjective wellbeing. Workers in health and education services also showed lower subjective wellbeing scores. Mutambudzi et al. [49]. Also noted that workers in health care, social care, and transportation were more likely to contract COVID-19 and more vulnerable to mental health impairment In addition, the current study reached a similar conclusion to previous studies that blue-collar, production, and transport workers suffered from worse subjective wellbeing during the pandemic [6,50,51]. It is increasingly being recognized that economic downturn results in financial and psychological pressures on individuals. Social distancing restrictions accompanying the pandemic have caused social isolation and created fears about financial insecurity. In addition, confirmed cases and increased deaths have exacerbated fear of infection and concerns about family members' health [52]. Many workers predicted drops in their future incomes due to uncertain conditions. The study findings emphasized that anxiety about economic status had negative effects on workers’ subjective wellbeing in line with Avdic et al. [39] who stated that fear of job loss and income drop during COVID-19 was associated with experiencing depression and anxiety. In addition, we found that workers' anxiety about catching COVID-19 was also positively correlated with poor subjective wellbeing in line with Kivi et al. [31] who highlighted that concern about COVID-19 infection is associated with low well-being scores.

The results concluded that COVID-19 changes in wages and employment status worsened workers' subjective wellbeing. Policy plans should promote the wellbeing workers in severely affected economic sectors. Social policies that allow access to health care and provide unemployment insurance benefits could protect workers' wellbeing. Furlough schemes accompanied by wage replacement plans are essential to reduce workers' vulnerability and preserve their livelihoods. Telemedicine provides a suitable alternative due to its cost-effectiveness and ability to expand access to specialized medical services in resource-constrained health systems, especially during crises [53,54].

The study made several contributions to previous literature. First, to the best of our knowledge, this is the first study to assess the implication of COVID-19 on workers’ subjective wellbeing in the MENA region. Second, the study depended on wide-ranging and representative data consisting of a set of harmonized surveys and covering four Arab countries. However, the study had the following limitations. First, the data was cross-sectional which hindered inferring a causal relationship between labour market outcomes and subjective wellbeing status during the pandemic. Second, the respondents were mobile phone users, and some population groups were missed, especially the poor and less educated workers who do not had mobile access. Third, there were other important variables affecting the workers' mental health were not included in the analysis due to data limitations, such as receiving COVID-19 vaccination, the death of a relative, experiencing depression, anxiety, suicidal ideation, sleep disorders, and perceived stress. Fourth, the survey did not measure subjective wellbeing status before the pandemic, which did not allow for a comparison of pre-and post-pandemic mental health scores. Finally, subjective wellbeing is related to personality traits, personal feelings should be considered, as optimistic personalities will have better psychological conditions than pessimistic personalities when facing the negative implications of the pandemic.

## 5 Conclusion

The COVID-19 pandemic has alarming implications on health and well-being. The pandemic outbreak has imposed strict lockdown measures, created mass unemployment, and negatively affected workers' subjective wellbeing. Using the latest data available in the MENA region, the study provided key insights into the impact of labour market changes on workers' subjective wellbeing. The study indicated that workers who lost their jobs or experienced income declines and wage delays had lower subjective wellbeing scores. The study also highlighted that workers with multiple vulnerabilities such as irregular work or non-institutional work were at higher risk of experiencing worse subjective wellbeing. Workers in accommodation and food services, information and communication, education, and health activities were most at risk of poor subjective wellbeing. Affected workers should be considered in intervention programs that address the psychological challenges caused by the pandemic.

## Ethics approval and consent to participate

We have used a secondary dataset (OAMDI, 2021). The data has been collected by Economic Research Forum (ERF) maintaining all ethical approval and consent to participate. This study received exempt status because it was a secondary analysis of an existing data set; We did not apply the questionnaire or collect the data in this study. We have not dealt with any human subject.

The datasets analyzed in the current study are available in “OAMDI, 2021. COVID-19 MENA Monitor Household Survey (CCMMHH), http://www.erfdataportal.com/index.php/catalog. Version 5.0 of the licensed data files; CCMMHH_Nov-2020-Aug-2021. Egypt: Economic Research Forum (ERF).” We obtained the dataset of this survey after emailing ERF. The full questionnaire of the CCMMHH is attached as a supplementary file.

## Availability of data and material

The datasets analyzed in the current study are available in “OAMDI, 2021. COVID-19 MENA Monitor Household Survey (CCMMHH), http://www.erfdataportal.com/index.php/catalog. Version 5.0 of the licensed data files; CCMMHH_Nov-2020-Aug-2021. Egypt: Economic Research Forum (ERF).” We obtained the dataset of this survey after emailing ERF. The full questionnaire of the CCMMHH is attached as a supplementary file.

## Funding

This work was supported and funded by the Deanship of Scientific Research at Imam Mohammad Ibn Saud Islamic University (IMSIU) (grant number IMSIU-RP23097).

## CRediT authorship contribution statement

**Mahmoud M. Abdelwahab:** Writing – review & editing, Writing – original draft, Supervision, Funding acquisition, Formal analysis, Data curation, Conceptualization. **Mohamed R. Abonazel:** Writing – review & editing, Writing – original draft, Visualization, Software, Methodology, Investigation, Formal analysis, Data curation, Conceptualization. **H.E. Semary:** Writing – review & editing, Validation, Supervision, Funding acquisition, Formal analysis, Data curation, Conceptualization. **Suzan Abdel-Rahman:** Writing – review & editing, Writing – original draft, Validation, Software, Methodology, Formal analysis, Data curation, Conceptualization.

## Declaration of competing interest

The authors declare the following financial interests/personal relationships which may be considered as potential competing interests:Mahmoud M. Abdelwahab reports financial support was provided by Imam Mohammad Ibn Saud Islamic University (IMSIU). If there are other authors, they declare that they have no known competing financial interests or personal relationships that could have appeared to influence the work reported in this paper.

## Figures and Tables

**Table A1:** Analysis variables and their definition

Variables	Definition
**Dependent variable**	
**Subjective wellbeing score**	Responses to the five statements (WHO-5 questionnaire) were aggregated to provide an overall assessment of subjective wellbeing status on a scale from 0 to 25 where 0 represents the worst status and 25 represents the best wellbeing status.
**Independent variables**	
**COVID-19 work outcomes**	
Temporary Layoff/suspension	labour market outcomes experienced by the workers i during the pandemic. Wage workers are asked whether they have experienced any of these changes during the COVID-19 pandemic or its related restrictions. Workers can mention multiple labour market changes simultaneously such as experiencing job loss and income reduction. Each outcome is binary variable that takes a value of 1 if encountered by the worker and 0 otherwise
Permanent Layoff/suspension	
Decline in working hours	
Decline in income	
Delay in wage payment	
unable to work from home	
Change in main job/activity	
**Work characteries before the pandemic**	
**Sector type**	Binary variable takes 1 for public sector workers in the public sector and 0 for those in private sector.
**Work stability**	Binary variable takes 1 for workers having regular jobs and 0 for those in irregular jobs.
**Social insurance**	Binary variable takes 1 for workers covered with social insurance and 0 otherwise.
**Work inside establishment**	Binary variable takes 1 for workers belonging to official establishment and 0 otherwise
**Occupation**	Categorical variable indicates the main occupation: Manager/professional, Technicians/associate professionals, Clerks/service workers, blue collar, production & transport workers)
**Main economic activity**	Categorical variable indicates the main economic activity. It includes broad categories, namely, Agriculture, fishing or mining, Manufacturing, Construction or utilities, Retail or wholesale, Transportation and storage, Accommodation and food services, Information and communication, Financial activities, Education activities, Health services, Other services
**Demographic variables**	
Age	Categorical variable includes three categories: younger adults (<30), adults (30–59), elderly (> 60)
Gender	Binary variable takes 1 if female and zero if male
Place of residence	Binary variable takes 1 if urban and 0 if rural
Education level	Categorical variable includes four categories: 1 for less than basic education and 2 for basic education and 3 for secondary education and 4 for higher education
Marital status	Categorical variable refers to the social status and includes three categories: never married, currently married, and divorced or widowed,
Household income quartiles	Household income quartile is determined based on the household's total income from all sources, including the respondent's income in February 2020. Household incomes are divided into four ranges (quartiles), each quartile containing 25% of observations. The lower quartile is denoted as the 1st quartile and the highest quartile is denoted as the 4th quartile.
Household size	Count variable indicates the number of individuals living in the same household
Presence of children	Binary variable takes 1 if the worker has children and 0 otherwise
**COVID-19 implications**	
**Social distancing variables**	
Staying at least meter away from others	Binary variables take 1 if the individual does the action and 0 otherwise
Wearing a mask outside home	
Washing hands more than before	
**Risks**	workers reported how worried they were about their economic situation on a scale from 1 to 4. 1 = Not at all worried, 2 = A little worried, 3 = Rather worried, and 4 = Very worried. workers reported how worried they were about catching COVID-19 infection on a scale from 1 to 4. 1 = Not at all worried, 2 = A little worried, 3 = Rather worried, and 4 = Very worried.
Worrying about economic situation	
Worrying about catching COVID-19	

**Table A2:** Distribution of workers experiencing COVID-19-induced labour outcomes according to their work characteristics.

	Temporary Layoff/suspension of Work	Permanent Layoff/suspension of Work	Decline in working hours	Decline in income	Delay in wage payment
**Type of sector**					
Public sector	7.4	6.7	21.9	8.8	18.1
Private sector	92.6	93.3	78.1	91.2	81.9
**Social insurance status**					
Insured	34	33.9	44.0	33.7	43.6
Uninsured	66	66.1	56.0	66.3	56.4
**Employment stability**					
Irregular	54.6	46.1	40.8	49.0	45.3
Regular	45.4	53.9	59.2	51.0	54.7
**Work inside establishment**					
Yes	47.7	51.2	60.6	55.7	58.4
No	52.3	48.8	39.4	44.3	41.6
**Occupation**					
Blue collar, production & transport	47.5	36.6	32.4	40.5	38.6
				
Clerks/service workers technicians & associate professionals	23.7	29.3	28.4	25.7	25.6
	17.5	21.8	21.1	18.5	20.4
Managers &Professionals	11.4	12.2	18.1	15.3	15.3
**Main economic activity**					
Agriculture, fishing &mining	9.6	7.8	6.8	7.5	7.6
Manufacturing	17.4	14.4	13.8	15.5	14.9
Construction & utilities	19.6	17.7	12.7	15.8	17.9
Retail & Wholesale	12.2	15.5	12.7	13.7	12.4
Transportation & storage	11.1	9.8	9.2	10.1	9.8
Accommodation & food services	10.8	11.5	7.0	9.4	7.6
Information & communication	2.1	3.0	2.8	1.8	2.7
Financial activities & real estate	2.1	3.0	7.0	6.3	3.0
Education	5.5	5.5	12.7	5.8	10.7
Health	2.8	3.5	3.4	2.9	4.7
Other services	6.8	8.3	11.9	11.1	8.7

**Table A3:** Distribution of workers by labour market changes and countries

Labour market changes	Country				Test statistic & P-value
	Jordan	Morocco	Tunisia	Egypt	
**Temporary Layoff/suspension of Work**					
Mentioned	260 (9.4)	349 (13.4)	1013 (29.9)	492 (27.5)	$\chi^{2}=557.47$
Not mentioned	2494 (90.6)	2264 (86.6)	2375 (70.1)	1299 (72.5)	<0.001***
**Permanent layoff/suspension of work**					
Mentioned	227 (8.2)	216 (8.3)	327 (9.7)	186 (10.4)	$\chi^{2}=10.31$
Not mentioned	2527 (91.8)	2397 (91.7)	3061 (90.3)	1605 (89.6)	0.036*
**Changes in number of working hours**					
Decline in working hours	421 (15.3)	288 (11.0)	777 (22.9)	1143 (40.3)	$\chi^{2}=950.32$ <0.001***
No changes in working hours	2155 (78.2)	2273 (87.0)	2358 (69.6)	1553 (54.8)	
Increase in working hours	178 (6.5)	52 (2.0)	253 (7.5)	138 (4.9)	
**Changes in hourly wage**					
Decline in income	332 (12.1)	190 (7.3)	623 (18.4)	875 (30.9)	$\chi^{2}=881.93$ <0.001***
Stable income	2380 (86.4)	2414 (92.4)	2661 (78.5)	1879 (66.3)	
Increase in income	42 (1.5)	9 (0.3)	104 (3.1)	80 (2.8)	
**Delay in wage payment**					
Mentioned	440 (16.0)	233 (8.9)	1102 (32.5)	460 (25.7)	$\chi^{2}=569.04$
Not mentioned	2314 (84.0)	2380 (91.1)	2286 (67.5)	1331 (74.3)	<0.001***
**No changes in employment status**					
Mentioned	1478 (53.7)	1653 (63.3)	1222 (36.1)	721 (40.3)	$\chi^{2}=520.69$
Not mentioned	1276 (46.3)	960 (36.7)	2166 (63.9)	1070 (59.7)	<0.001***
**Mean SWB score**	9.54	10.81	9.61	10.39	F=124.17 <0.001***

***P < 0.001, **p < 0.01, *p < 0.05.
