# Supplementary material for: Implications of labour market disruptions on subjective wellbeing during the COVID-19 pandemic in MENA countries
Source: Heliyon. 2024 Feb 12;10(4):e25665. doi: 10.1016/j.heliyon.2024.e25665 (PMC10881308; doi:10.1016/j.heliyon.2024.e25665)
Supplement: Multimedia component 1 [file mmc1.pdf]

COVID-19 ECONOMIC IMPACT SURVEY

					Notes
Tel_op	What is the type of operator?	**1= Operator Company:** Number [ ]<br>2= **Operator Company:** Number [ ]<br>3= **Operator Company:** Number [ ]<br>4**= Any other operator (specify)** : Number [ ], specify: text [ ]			Need country-specific adaptation
COR1	Telephone #				bold if read it
COR2_1	Result of call	Phone disconnected/ busy	1		Try for up to 3 times
		Not in service	2		
		Did not answer	3		Try for up to 3 times
		Picked up and refused	4		
		Incomplete and refused	5		
		Incomplete and call returned	6		Try for up to 3 times
		Complete	7		
		Not eligiable	8		
Time_1	Time for first result of call				
Date_1	Date for first result of call				
COR2_2	Result of call	Phone disconnected/ busy	1		Try for up to 3 times
		Not in service	2		
		Did not answer	3		Try for up to 3 times
		Picked up and refused	4		
		Incomplete and refused	5		
		Incomplete and call returned	6		Try for up to 3 times
		Complete	7		
		Not eligiable	8		
Time_2	Time for second result of call				
Date_2	Date for second result of call				
COR2_3	Result of call	Phone disconnected/ busy	1		Try for up to 3 times
		Not in service	2		
		Did not answer	3		Try for up to 3 times
		Picked up and refused	4		
		Incomplete and refused	5		
		Incomplete and call returned	6		Try for up to 3 times
		Complete	7		
		Not eligiable	8		
Time_3	Time for third result of call				
Date_3	Date for third result of call				
**Consent**					
COR3	**Good morning/ afternoon/ evening, I am .... from Phi (a research agency). We spoke to you two months ago about the economic impact of the COVID-19 outbreak, and we would like to ask you few questions again today.**<br><br>**This survey will take approximately 15 minutes to complete.**<br>**We will ask about your economic activities prior to being affected by COVID-19 and how the outbreak impacted you.**<br><br>**You may also be asked personal information that is important for the results of the study, some of which might make you feel anxious or embarrassed. You will also have a chance to share your contact information at the end of the survey, in case you are interested in participating in a follow-up survey. Please note that this information will be kept strictly confidential. You can choose to answer follow-up surveys every two months for up to a total of four interviews.**<br><br>**Your participation is completely voluntary. You can choose not to take part. You can agree to take part and later change your mind without any Consequences. Your refusal to participate will not result in any consequences or any loss of benefits that you are otherwise entitled to receive. We will compensate your time and participation with phone credits worth XXXXXX from your phone provider .Otherwise you will receive no direct benefits from participating in the study.**<br><br>**If you have questions, concerns, or complaints, or think the research has hurt you, please contact [XXXXXXX].**<br><br>**Do you agree to participate in this survey?**		1=Yes; 2=No	If no (=2), end interview	Add incentives
COR4	**Do you agree to be recorded for quality control purposes?**		1=Yes; 2=No		

**Education and Children**					
COR16	**How many children who are enrolled in school live in your current household?**	Number [ ]		If 0, skip next question	
COR17	**What are children in the household doing to spend time on education?<br>(Enumerator: read all responses one by one, and mark all that apply)**	**Educational television**	1		
		**Online education**	2		
		**Official educational content - written materials**	3		
		**I was helping or teaching them**	4		
		**Others in my household were helping or teaching them**	5		
		**In person school**	6		
		**Other**	7		
		**None of the above**	8		
**Labour market status**					
COR20	**What was your main job/activity in the past month?<br>Enumerator:<br>1- if answer don't work, then ask if he/she is looking for a job and ready to work, to confirm if he/she will be code 7<br>2- working student: choose based on the main activity**	Farmer (owns a farm/self-employed on a farm)	1		
		Business owner/self employed (but not a farmer)	2		
		Unpaid family worker on a farm	3		
		Unpaid family worker (but not a farmer)	4		
		Wage worker for Government / Public sector	5		
		Wage Worker for a private sector /NGO	6		
		Unemployed and looking for work	7		
		Housewife	8		
		Full Time Student	9		
		Retired	10		
		Other, not employed and not looking for work (e.g. taking care of family members)	11		
**Food Security**					
COR21	**In the past 7 days, have you or any household member experienced any of the following?<br>(Enumerator: read all responses one by one, and mark all that apply)**	**Difficulties in going to food markets due to mobility restrictions imposed by government/closures**	1		
		**Unable to buy the amount of food we usually buy because of shortages of food in markets**	2		
		**Unable to buy the amount of food we usually buy because the price of food increased**	3		
		**Unable to buy the amount of food we usually buy because our household income has dropped**	4		
		**Had to reduce the number of meals and/or the portion of each meal we would usually eat**	5		
		**None**	6		
**Income**					
COR23	**Did your household's total monthly income increase or decrease last month compared to Feb 2020?**	Decreased by more than 25%	1		
		Decreased by 1-25%	2		
		Stay the same	3		
		Increased by 1-25%	4		
		Increased by more than 25%	5		
**Safety net**					
COR25	**Have you received any food, cash or other support from the government in the past month that you do NOT usually receive? If so, which type of support?<br>(Enumerator: read all responses one by one, and mark all that apply)**	**Food**	1		May need adaptation, although broad categories helpful
		**Cash**	2		
		**PPEs (Personal Protective Equipment, such as gloves, masks, soap, sanitizer)**	3		
		**Other (specify)**	4		
		**None**	5		
COR26	**Have you received any food, cash or other support from anyone else in the past month, that you do NOT usually receive? If so, from which source?<br>(Enumerator: read all responses one by one, and mark all that apply)**	**Church/mosque**	1		
		**Relatives in country**	2		
		**Relatives outside the country**	3		
		**Member of parliament or other politicians**	4		
		**NGO/CSO**	5		
		**Other (specify)**	6		
		**None**	7		
COR27	**Did you need to resort any of these coping strategies In the past month?<br>(Enumerator: read all responses one by one, and mark all that apply)**	**Taking money out of Savings**	1		
		**Family, relatives, or friends in (country)**	2		
		**Family, relatives, or friends abroad**	3		
		**Going back to your village or family?**	4		
		**Borrowing from a bank, employer, or private lender**	5		
		**Selling assets**	6		
		**Some other source (specify)**	7		
		**None**	8		

**Employment Detection**					
COR28	**In the past 7 days, did you spend at least one hour working? Please consider day labor, work for wages or in-kind, and working on your own account or your own business, including an agricultural business (farm).**		1=Yes; 2=No	Skip next question if no	
COR29	**In the past 7 days, how many hours did you work?**		[__|__] hours	Finish employment detection section for this group, go to COR34	
COR30	**Were you attached to a job in the past 7 days but were temporarily absent from it?**		1=Yes; 2=No	if yes, Skip to COR34	
COR31	**In the past 7 days, were you wanting, willing and available to work?**		1=Yes; 2=No	Skip next two, if no	
COR32	**In the past four weeks, did you actively search for work?**		1=Yes; 2=No	If yes skip next	
COR33	**Why did you not search?**	Believe there are no jobs at all	1		
		Unable to search due to COVID-19	2		
		No suitable job	3		
		Lack of personal connections (wasta)	4		
		Because of studying	5		
		Family responsibilities	6		
		Other (specify)	7		
		Don't know	8		
**Risks**					
COR34	**How worried are you about being infected with COVID-19? On a scale from 1 to 4 from not at all worried (1) to very worried (4) where would you put yourself? (Enumerator: read the responses)**	**Not at all worried**	1		
		**A little worried**	2		
		**Rather worried**	3		
		**Very worried**	4		
		**I had it already (don't read)**	5		
COR35	**How worried are you about the economic situation? On a scale from 1 to 4 from not at all worried (1) to very worried (4) where would you put yourself? (Enumerator: read the responses)**	**Not at all worried**	1		
		**A little worried**	2		
		**Rather worried**	3		
		**Very worried**	4		
**Mental Health**					
	**Over the past 2 weeks…**	**(Responses repeat for each question)**	**1. All of the time**		
COR36	**I have felt cheerful and in good spirits. (Enumerator: read the responses)**		**2. Most of the time**		
COR37	**I have felt calm and relaxed. (Enumerator: read the responses)**		**3. More than half the time**		
COR38	**I have felt active and vigorous. (Enumerator: read the responses)**		**4. Less than half the time**		
COR39	**I woke up feeling fresh and rested. (Enumerator: read the responses)**		**5. Some of the time**		
COR40	**My daily life has been filled with things that interest me. (Enumerator: read the responses)**		**6. At no time**		
**Social Distancing**					
COR41	**Do you try to stay at least one meter away from other people when outside the house?**		1=Yes; 2=No		
COR42	**Do you wear a mask when outside the house?**		1=Yes; 2=No		
COR43	**Do you wash your hands with soap more often than you did before COVID-19?**		1=Yes; 2=No		
	[THEN MOVE TO BUSINESS QUESTIONNAIRE IF COR20=2]				
	[MOVE TO WORKER QUESTIONNAIRE IF COR20=5,6]				
	[MOVE TO FARMER QUESTIONNAIRE IF COR20=1]				
	[THEN MOVE TO TRACKING QUESTIONNAIRE IF MALE and COR20=3,4, 7,8,9,10,11]				
	[THEN MOVE TO WOMEN QUESTIONNAIRE IF FEMALE and COR20=3,4, 7,8,9,10,11]				

COVID-19 ECONOMIC IMPACT SURVEY

**Worker module**				
WOR1	Which activity best describes your main job/activity the past month?	Agriculture, fishing or mining	1	
		Manufacturing	2	
		Construction or utilities	3	
		Retail or Wholesale	4	
		Transportation and storage	5	
		Accomodation and food services	6	
		Information and communication	7	
		Financial activities or real estate	8	
		Education	9	
		Health	10	
		Other services (specify)	11	
**Occupation**				
WOR2	The past month, what was your occupation?	Manager/professional	1	
		Technicians/associate professionals	2	
		Clerks/service workers	3	
		Blue collar, skilled agricutlural, production and transport	4	
WOR3	The past month, what was the stablility of your employment status?<br>(Enumerator: read the responses)	Regular (permanent or temporary)	1	
		Irregular (causal, seasonal, or intermittent)	2	
WOR4	Do you have social insurance in your job?		1=Yes; 2=No	
WOR5	Do you work inside an establishment?		1=Yes; 2=No	
WOR6	How many paid employees does the business you work for have currently (both full-time and part-time, including yourself)?	[__|__|__] workers		
WOR8	In the past month, what was your personal net monthly wage (including overtime, bonuses)? ENUMERATOR: If don't know monthly wages but know for another time frame, ask questions on time to figure out monthly wages. Use 99998 if don't know, 99996 if refuse	[__|__|__] wage in LCU		
**COVID -19 questionnaire**				
WOR9	As of today, what is the status of the business you worked for the past month?	Temporarily closed due to government mandate related to the	1	
		Chose to temporarily close due to other challenges related to	2	
		Temporarily or permanently closed due to factors unrelated to	3	
		Permanently closed due to challenges related to the COVID-1	4	
		Open with the same business hours	5	
		Open but reduction in business hours due to government mandate related to the COVID-19 outbreak	6	
		Business chose to reduce number of business hours	7	
		Do not know	8	

WOR10	In the last 60 days, has the business you work for been facing any of the following challenges due to the coronavirus/COVID-19 outbreak and related restrictions? (Enumerator: read all responses one by one, and mark all that apply)	Difficulties in accessing customers due to mobility restrictions imposed by government	1	
		Loss in demand due to other reasons (e.g., regular customers can no longer afford our products or services or have cancelled orders)	2	
		Difficulties in accessing suppliers due to mobility restrictions imposed by government	3	
		Reduction in the availability and/or price increases for the main inputs	4	
		Difficulties with worker absenteeism arising from transport problems/mobility restrictions imposed by the government	5	
		Other (specify)	6	
		No particular challenge, things have proceeded as normal	7	
WOR11	In the last 60 days, have you experienced any of the following because of Covid-19/coronavirus or related restrictions? (Enumerator: read all responses one by one, and mark all that apply)	Temporary layoff/suspension of work (without pay)	1	
		Permanent layoff /suspension of work	2	
		WOR11_3: Change in number of working hours	1) Decrease by more than 25%<br>2) Decrease by 1- 25%<br>3) Stay the same<br>4) Increase by 1-25%<br>5) Increase by more than 25%	
		WOR11_4: Change in hourly wage, piece rate, or salary	1) Decrease by more than 25%<br>2) Decrease by 1- 25%<br>3) Stay the same<br>4) Increase by 1-25%<br>5) Increase by more than 25%	
		Delay in wage payment.	5	
		Hired	6	
		No, none (Don't read)	7	
WOR12	Are you able to work from home?		1=Yes; 2=No	Skip next if answer yes
WOR13	Why are you not able to work from home?	Not allowed	1	
		Lack technology	2	
		Lack internet connection	3	
		Caregiving responsibilities	4	
		Not possible to do job off work site	5	
		Other (specify)	6	
[THEN MOVE TO WOMEN QUESTIONNAIRE IF FEMALE, TRACKING IF MALE]				

COVID-19 ECONOMIC IMPACT SURVEY

Farmer module

Farmers

FAR1	**What is the most important crop/livestock or dairy products for your household?**	Cereals	1	USED ICC CROP CODES
		Vegetables and melons	2	
		Fruits and nuts	3	
		Poilseed crops and oleaginous fruits	4	
		Root/tuber crops with high starch or inulin content	5	
		Stimulant, spice and aromatic crops	6	
		Leguminous crops	7	
		Sugar crops	8	
		Livestock or Dairy Products	9	
		Other crops (specify)	10	
Enumerator READ:	**The following questions refer to the crop you mentioned in FAR1**			
FAR2	**In which phase of the crop cycle are you?**	Land preparation	1	
		Planting	2	
		Crop on the farm: Applying inputs, weeding	3	
		Harvesting	4	
		Selling	5	
		None of the above (off-season)	6	Go to FAR8
FAR3	**Relative to the same season in 2019, how many days did you and your household members spend on this activity on your farm? (Enumerator: read the responses)**	I was not allowed to go to the farm this year	1	
		Much fewer days, lowest number of days in past 5 years	2	
		Fewer days	3	
		About the same	4	
		More days	5	
		Many more days, highest number of days in past 5 years	6	
		Not applicable	7	
FAR4	**Relative to the same season in 2019, how many days did you hire workers to work on this activity on your farm? (Enumerator: read the responses)**	I was not allowed to hire other people on my farm this year.	1	
		Much fewer days, lowest number of days in past 5 years	2	
		Fewer days	3	
		About the same	4	
		More days	5	
		Many more days, highest number of days in past 5 years	6	
		Not applicable	7	

FAR5	**Relative to the same season in 2019, how many days did you and your household members spend on this activity on other people's farms (including plantations)?<br>(Enumerator: read the responses)**	I was not allowed to work on other people’s farm this year	1	
		Much fewer days, lowest number of days in past 5 years	2	
		Fewer days	3	
		About the same	4	
		More days	5	
		Many more days, highest number of days in past 5 years	6	
		Not applicable	7	
FAR6	**Relative to the same season in 2019, how many seeds and inputs (e.g. fertilizer, chemicals, animals' feed) have you used (do you plan to use) for your farm for this crop?<br>(Enumerator: read the responses)**	Not allowed to go buy inputs; inputs are not available	1	
		Much less, lowest amount in past 5 years	2	
		Less	3	
		About the same	4	
		More	5	
		Much more, highest amount in past 5 years	6	
		Not applicable	7	
FAR7	**Relative to the same season in 2019, how much have you harvested (do you expect to harvest) for your farm for this crop/ products?<br>(Enumerator: read the responses)**	Not allowed to go harvest.	1	
		Much less, lowest amount in past 5 years	2	
		Less	3	
		About the same	4	
		More	5	
		Much more, highest amount in past 5 years	6	
		Not applicable	7	
FAR8	**Relative to the same season in 2019, how are /do you expect prices to change for this crop/ product?<br>(Enumerator: read the responses)**	Much lower, lowest price in the last 5 years.	1	
		Lower	2	
		About the same	3	
		Higher	5	
		Much higher, highest price in the last 5 years	6	
FAR9	**Are you/do you expect to be able to sell your crop/ product in the locations/markets where you usually sell it?**	Yes	1	
		No, but I expect to find other channels to sell all my crop/ product	2	
		No, I will likely not be able to sell all my crop/ product	3	
		No, I will likely not be able to sell any of my crop/ product	4	
		Not applicable	7	
[THEN MOVE TO WOMEN QUESTIONNAIRE IF FEMALE, TRACKING IF MALE]				

COVID-19 ECONOMIC IMPACT SURVEY

Business module

HHE1	**Which activity best describes your main job/activity the past month?**	Agriculture, fishing or mining	1	
		Manufacturing	2	
		Construction or utilities	3	
		Retail or Wholesale	4	
		Transportation and storage	5	
		Accomodation and food services	6	
		Information and communication	7	
		Financial activities or real estate	8	
		Education	9	
		Health	10	
		Other services (specify)	11	
**Operations**				
HHE2	**How many workers did your business have the past month? (both full-time and part-time, including family members but excluding yourself)**	[__|__|__] workers		
HHE4_1	**did your sales/revenues increase or decrease in the last 60 days compared to your sales/revenue in the same period at 2019?**	increased	1	skip next question if not change
		decreased	2	
		no change	3	
HHE4_2	**what is the percentage of this increase or decrease in your sales/revenues?**	[__|__] percent		
**COVID -19 questionnaire**				
HHE5	**In the last 60 days, has your business been facing any of the following challenges due to the coronavirus/COVID-19 outbreak and related restrictions?<br>(Enumerator: read all responses one by one, and mark all that apply)**	**Difficulties in accessing customers due to mobility restrictions imposed by government**	1	
		**Loss in demand due to other reasons (e.g., regular customers can no longer afford our products or services or have cancelled orders)**	2	
		**Difficulties in accessing suppliers due to mobility restrictions imposed by government**	3	
		**Reduction in the availability and/or price increases for the main inputs**	4	
		**Difficulties with worker absenteeism**	5	
		**Difficulties tending to my business because I have to take care of a family member (e.g. children, sick relative, etc)**	6	
		**other (specify)**	7	
		**No particular challenge, things have proceeded as normal**	8	

HHE6	**What is the current status of your business? (Note: if the business is closed to the public but operates it should be considered open)**	Temporarily closed due to government mandate related to the COVID-19 outbreak	1	
		Chose to temporarily close due to other challenges related to the COVID-19 outbreak	2	
		Temporarily or permanently closed due to factors unrelated to the COVID-19 outbreak	3	
		Permanently closed due to challenges related to the COVID-19 outbreak	4	
		open with the same business hours	5	
		open but reduction in business hours due to government mandate related to the COVID-19 outbreak	6	
		Open but reduction in business hours because I chose to	7	
		Do not know	8	
HHE7	**Was the business closed by government mandate?**		1=Yes; 2=No	Only ask if business is closed
HHE8	**In the last 60 days, how many of your workers experienced any of the following as a result of the coronavirus/covid-19 outbreak and related restrictions?**	**HHE8_1: Temporary layoff/suspension of work (without pay)**	[__|__|__] workers	Skip next question if business closed
		**HHE8_2: Permanent layoff/suspension of work (without pay)**	[__|__|__] workers	
		**HHE8_3: Reduction in earnings from this work or delays in wage payment**	[__|__|__] workers	
		**HHE8_4: Hired additional workers**	[__|__|__] workers	
HHE9	**Under current conditions, for how much longer do you think you will be able to keep your business open?**	Less than 2 weeks	1	
		Between 2 and 4 weeks	2	
		Between 1 and 2 months	3	
		Between 2 and 6 months	4	
		More than 6 months	5	
		Don't know (uncertain)	6	
HHE10	**How has your business adjusted its business model to reduce being directly in physical proximity with customers? (Enumerator: read all responses one by one, and mark all that apply)**	**Use of phone for marketing, placing order etc.**	1	
		**Use of Internet, online social media, specialized apps or digital platforms**	2	
		**Switched product**	3	
		**Other (specify)**	4	
		**No change in business model**	5	
**Policy response**				
HHE11	**Have you applied for or are you currently receiving any government programs to support businesses like yours? (Enumerator: read all responses one by one, and mark all that apply)**	**Business loans**	1	
		**Loan payment deferrals**	2	
		**Partial or total salary subsidies**	3	
		**Cash transfers of unemployment benefits**	4	
		**Rental or utilities subsidies or deferrals**	5	
		**Subsidized provision of specific products, inputs or services**	6	
		**Reduction or delay in taxes**	7	
		**I haven't applied for any programs**	8	

HHE12	**What would be the most needed policy to support your business over the COVID-19 crisis?**	Business loans	1	
		Loan payment deferrals	2	
		Partial or total salary subsidies	3	
		Cash transfers or unemployment benefits	4	
		Rental or utilities subsidies or deferrals	5	
		Subsidized provision of specific products, inputs or services	6	
		Reduction or delay in taxes	7	
		Others (specify)	8	
		Nothing	9	
HHE13	**Why have you not applied to any government programs? (Enumerator: read all responses one by one, and mark all that apply)**	I am not aware of any such programs	1	Skip if did apply
		It requires internet/smart phone and I do not have one	2	
		Even if I apply, I don't think I will get support from these programs	3	
		I will need to pay a bribe to apply to these programs	4	
		Others (specify)	5	
		Nothing	6	
**Future**				
HHE14	**In the next 6 months, how many of your workers do you expect will experience any of the following as a result of the coronavirus/covid-19 outbreak and related restrictions?**	**HHE14_1: Temporary layoff/suspension of work (without pay)**	[__|__|__] workers	Skip if business not open
		**HHE14_2: Permanent layoff/suspension of work (without pay)**	[__|__|__] workers	
		**HHE14_3: Reduction in earnings from this work or delays in wage payment**	[__|__|__] workers	
		**HHE14_4: Hired additional workers**	[__|__|__] workers	
HHE15_1	**Over 2021, do you expect any change(increase/decrease) in your business' total sales comparing to 2019?**	increased	1	skip next question if not change
		decreased	2	
		no change	3	
HHE15_2	**What is the percentage of change (increase/decrease) that you expect in your business' total sales?**	[__|__] percent		
HHE16	**When are you expecting that this business will resume operations?**	Less than 2 weeks	1	Skip if business open
		Between 2 and 4 weeks	2	
		Between 1 and 2 months	3	
		Between 2 and 6 months	4	
		More than 6 months	5	
		Never	6	
		Don’t know (uncertain)	7	

[THEN MOVE TO WOMEN QUESTIONNAIRE IF FEMALE, TRACKING IF MALE]

COVID-19 ECONOMIC IMPACT SURVEY

Women module				
WOM1	**Does your household include children under age 18?**	1=Yes; 2=No		If No -->WOM5
WOM2	**On a typical day in the past week, how many hours did you spend taking care of children (exclusively or while doing other things)?**	Hours [_]		
WOM3	**How does the time you spent caring for children in the past week compare to the time you spent in a normal week in February 2020?<br>(Enumerator: read the responses)**	**More than usual**	1	
		**Same**	2	
		**Less than usual**	3	
WOM5	**On a typical day in the past week, how many hours did you spend doing housework (e.g. cooking, cleaning, washing dishes, shopping for daily necessities)?**	Hours [_]		
WOM6	**How does the time you spent doing housework in the past week compare to the time you spent in a normal week in February 2020?<br>(Enumerator: read the responses)**	**More than usual**	1	
		**Same**	2	
		**Less than usual**	3	
WOM7	**In the past seven days, which of the following activities did you spend time doing for your household?<br>(Enumerator: read all responses one by one, and mark all that apply)**	**Cooking, serving meals, washing dishes**	1	
		**Cleaning, doing other housework**	2	
		**Doing house repairs**	3	
		**Shopping or transporting members living with you**	4	
		**Feeding, bathing, playing with or putting to sleep children aged 5 or less**	5	
		**Tutoring, playing with or other care for children aged 6-17 living with you**	6	
		**Caring for ill or dependent adult members living with you**	7	
		**None**	8	

COVID-19 ECONOMIC IMPACT SURVEY

Tracking module

TR1	**We have now come to the end of our interview. Thank you for your participation. We would like to offer you airtime for sharing your opinions with us.**			
TR2	**Should I send the airtime to this phone number?**	Yes, this number	1	Skip next two questions if yes
		No, another number	2	
TR3	**If another number what is that number?**			
TR4	*Enter phone number second time*<br>[survey validation that they match]			
TR5	**Which network do you use with that number so that we can send the correct airtime? (Enumerator: single response only)**	**1= Operator Company:** Number [ ]<br>2= **Operator Company:** Number [ ]<br>3= **Operator Company:** Number [ ]<br>4**= Any other operator (specify)** : Number [ ], specify: text [ ]		Needs adaptation
TR6	**Read: We would like to be able to contact you again in the future to participate in additional research on the effects of covid-19 in [Country]. If you agree we will keep your contact details along with your survey data and may contact you in the next few months. We will not share your contact information with anyone other than staff members and researchers from [firm]. You may also decline participation in any or all future studies at no personal cost.**			
TR7	**Would you be willing to be contacted again in the future to participate in additional research on the effects of covid-19 in [Country]?**	1=Yes; 2=No	If No, end	
TR8	**What is your full name?**			
TR9	**Is this the correct phone number in case we want to ask some follow up questions?**	1=Yes; 2=No	If no, skip to TR 12	
TR10	*If yes*<br>Confirm RDD number.			
TR11	*Enter phone number twice*<br>[survey validation that they match]			
TR12	*If no*<br>Enter correct phone number twice.			
TR13	*Enter phone number twice*<br>[survey validation that they match]			
TR14	**Do you have a second phone number we could reach you on?**	1=Yes; 2=No	If no, skip to TR 17	
TR15	**If yes enter phone number twice.**			
TR16	*Enter phone number twice*<br>[survey validation that they match]			
TR17	**Can you please provide contact details of one person who would know how to get in touch with you if you move or lose your phone and we wish to re-contact you for clarifications or to participate in additional research?**	1=Yes; 2=No	If no, skip to TR 22, Thank and end interview	
TR18	If yes enter name of person and then phone number of this person twice. Name 1			
TR19	Name 1 [survey validation that they match]			
TR20	Enter phone number of [Name 1] twice. Phone number			
TR21	*Enter phone number twice*<br>[survey validation that they match]			
TR22	**Thank you for your participation in our survey. Have a good day.**	[End survey]		
